# Supplementary figures and images for: Histone demethylase JMJD2D protects against enteric bacterial infection via up-regulating colonic IL-17F to induce β-defensin expression
Source: PLoS Pathog. 2024 Jun 21;20(6):e1012316. doi: 10.1371/journal.ppat.1012316 (PMC11221690; doi:10.1371/journal.ppat.1012316)

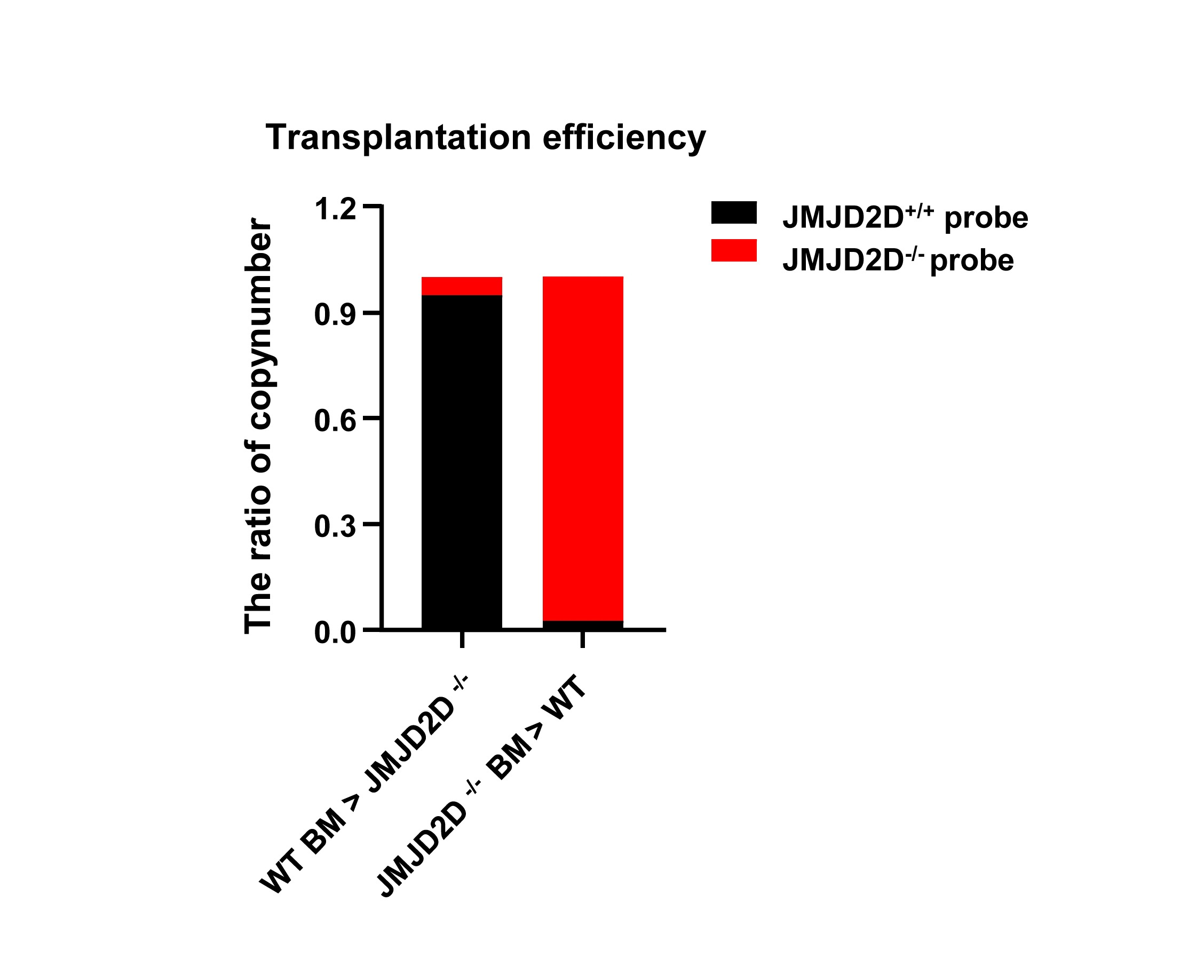

Supplement: S1 Fig — The efficiency of bone marrow transplantation was assessed by designing distinct probes targeting both wild-type and knockout alleles of JMJD2D in murine peripheral blood genomic DNA via digital PCR. n = 6. (TIF) [file ppat.1012316.s001.tif]

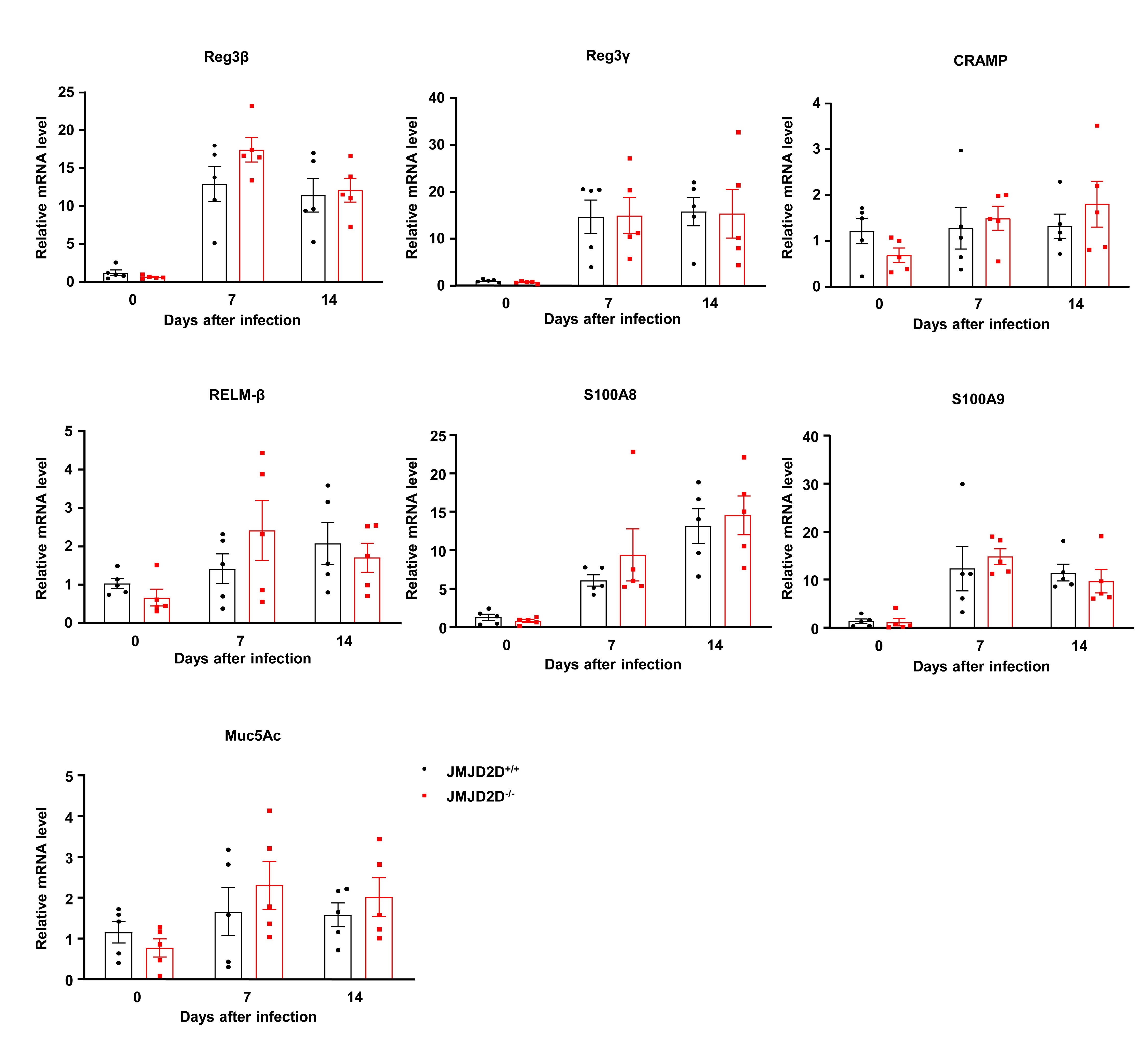

Supplement: S2 Fig — There is no significant difference in the expression of Reg 3β, Reg 3γ, CRAMP, RELM-β, S100A8, S100A9, and Muc5Ac in the colonic epithelial cells of wild-type and JMJD2D-/- mice after C. rodentium infection. Results are representative of three independent experiments. (TIF) [file ppat.1012316.s002.tif]

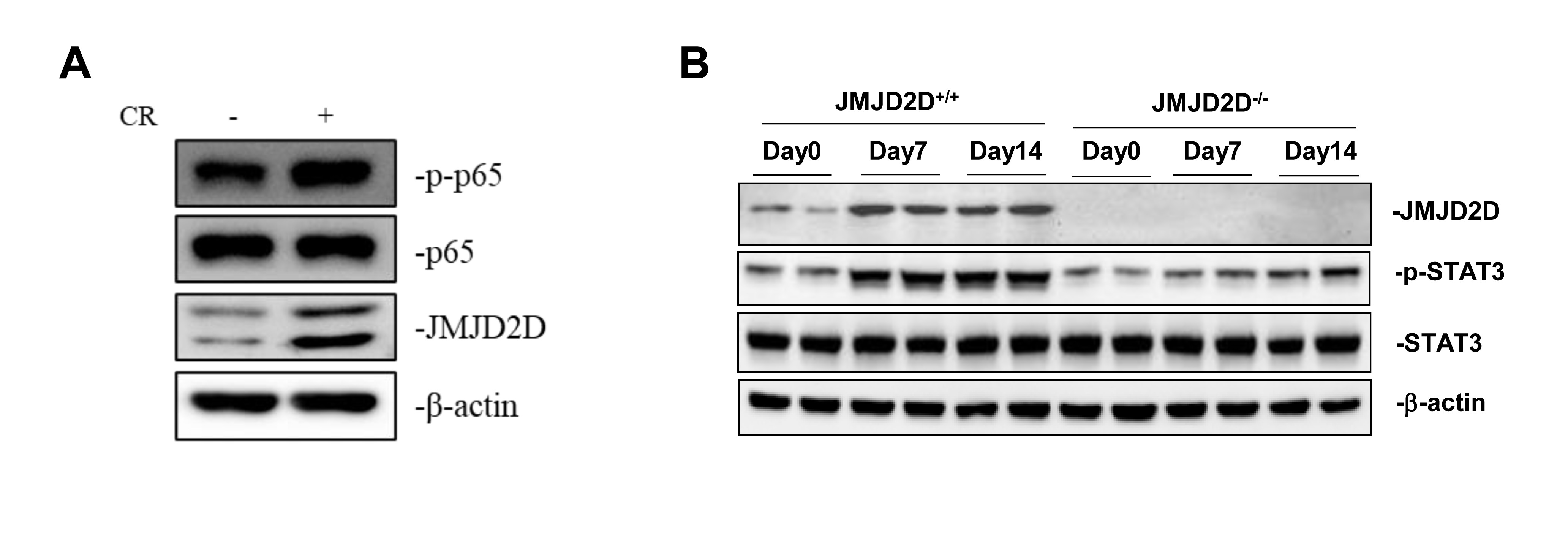

Supplement: S3 Fig — (A) Heat-killed C. rodentium treatment could activate NF-κB signaling by increased phosphorylation of p65. (B) STAT3 phosphorylation was impaired in the colonic epithelial cells of JMJD2D-/- mice after C. rodentium infection. (TIF) [file ppat.1012316.s003.tif]

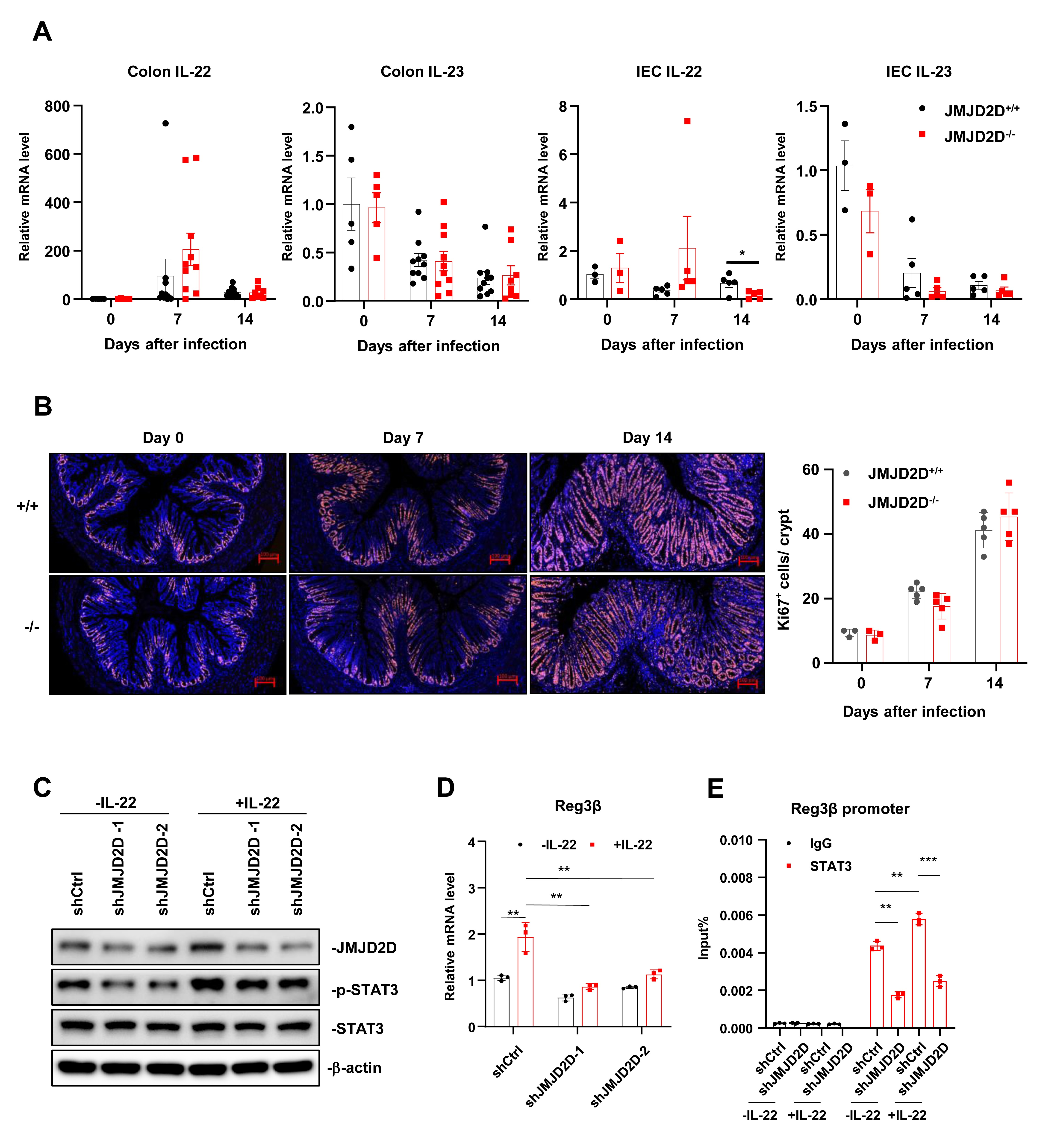

Supplement: S4 Fig — (A) No significant differences were observed in the expression of IL-22 and IL-23 in the colons of wild-type and JMJD2D-/- mice on days 7 and 14 after C. rodentium infection. On day 14 after C. rodentium infection, JMJD2D deficiency led to impaired IL-22 expression in colonic epithelial cells. No significant difference was observed in IL-23 expression in colonic epithelial cells of wild-type and JMJD2D-/- mice infected with C. rodentium. (B) Ki67 staining revealed comparable numbers of Ki67+ cells per crypt between wild-type mice and JMJD2D-/- mice without or with C. rodentium infection. (C, D) JMJD2D knockdown reduced IL-22-induced STAT3 phosphorylation (C) and Reg3β expression and (D). (E) JMJD2D knockdown reduced the recruitment of STAT3 on the Reg3β promoter without or with IL-22 treatment. Results are representative of three independent experiments. **P<0.01; ***P<0.001. (TIF) [file ppat.1012316.s004.tif]
