# Supplementary material for: Histone demethylase JMJD2D protects against enteric bacterial infection via up-regulating colonic IL-17F to induce β-defensin expression
Source: PLoS Pathog. 2024 Jun 21;20(6):e1012316. doi: 10.1371/journal.ppat.1012316 (PMC11221690; doi:10.1371/journal.ppat.1012316)
Supplement: Attachment — Submitted filename: Rebuttal letter-Yu.doc [file ppat.1012316.s006.doc]

May 10, 2024

Michael Malim

Editor-in-Chief, PLOS Pathogens

On behalf of the Editorial Board

Dear Malim,

Thank you for handling and examining our manuscript entitles “Histone demethylase JMJD2D protects against enteric bacterial infection via up-regulating colonic IL-17F to induce β-defensin expression” (Manuscript Number: PPATHOGENS-D-23-02288). We also thank the reviewers’ comments on our manuscript. In response to reviewers’ comments, we have performed the suggested experiments and substantially revised our manuscript. We believe that we have adequately addressed reviewer’ comments, please refer to the point-to-point responses to the comments for detail changes.

We hope this revised version of our manuscript is now appropriate for PLOS Pathogens.

Sincerely yours,

Chundong Yu, Ph.D.

Professor

School of Life Sciences

Xiamen University

Xiamen, Fujian 361102, China

Reviewer's Responses to Questions

**Part I - Summary**
Please use this section to discuss strengths/weaknesses of study, novelty/significance, general execution and scholarship.

Reviewer #1: In this new article, Zhang et al identified a new role of JMJD2D in colonic epithelial cells to help defend against C. rodentium infection. They then demonstrate that JMJD2D in colonic epithelial cells increases STAT3 activity to work together for up-regulating IL-17F, thereby increasing beta-defensin expression.
 The experimental design is logic and well considered. The results are clear and strong to support the major conclusions, which is of interest to the related research fields.

**Response: Thank you for your comments.**

Reviewer #2: How the gene regulatory machinery, including histone modifying enzymes, are involved in innate immunity processes is an important area of research with many implications. In the study by Yong Zhang et al, the team address the role of H3K9m2/3 demethylase JMJD2D (also known as KDM4D) in this process. They focus on the Citrobacter rodentium infection model in the mouse that bears relevance to understanding infection in human, too, e.g. infection by pathogenic E. coli. This study complements previous work by the team that examined the role of JMJD2D in experimental colitis in the mouse.
 In this paper the authors show that deletion of JMJD2D causes increased pathology on Citrobacter rodentium infection (Fig. 1) and indicate a role of JMJD2D in non-bone marrow-derived cells in this protective function, presumably the intestinal epithelium (Fig.2). They implicate a role of JMJD2D in regulating specific anti-bacterial β-defensins and IL-17F expression in colonic epithelial cells in this protective function (Fig.3). They show in cells in culture that IL-17F is required for the expression of several defensins (Fig.4). Importantly, ectopic IL-17F expression in JMJD2D–knockdown cells or treating these cells with recombinant IL-17F rescued the expression of β-defensin-1, β-defensin-3, and β-defensin-4. The authors provide evidence that heat-killed C. rodentium treatment of cells in culture causes JMJD2D to cooperate with STAT3 to induce IL-17F expression by demethylating H3K9me3 on the IL-17F promoter (Fig.5). They provide data supporting that the demethylase activity of JMJD2D is required for the induction of IL-17F and β-defensins (Fig.6).
 This is a fairly clear-cut and interesting study that ads to the picture how histone modifying factors are involved in innate immunity processes controlling infection. The data are convincing, the presentation is logic and I see now major issue.

**Response: Thank you for your comments.**

Reviewer #3: This manuscript nicely describes work on how a Histone Demethylase (JMJD2D) is induced upon infection/inflammation (not necessarily novel), and that it likely plays a role in controlling epithelial IL-17F, which the authors suggest is due to controlling phospho-Stat3 and/or Stat3-JMJD2D interaction near the IL-17F locus. Subsequently, the IL17F controls B-Defensin expression, which would act as antimicrobials to control a bacterial infection. Together, the authors propose JMJD2D is required for the demethylation of H3K9 to allow for gene expression (Il17F) activation. While both the in vitro and in vivo data is strong and nicely presented, there are a few gaps that are not addressed.

Strengths: Clear phenotype in the KO during Citro – weight loss and bacterial burden are clearly affected, which correlates with the histology. In addition, Figs 5&6 nicely show some mechanistic work that is quite strong.

Weakenesess: the bone marrow exps lack the data that shows that the bone marrow transplant was successful (and what percentage). In addition, the biological data in the BM experiments is less convincing than the full KO. The proposed mechanisms (reduced pStat3) should be evaluated in the in vivo exps. Normally, I would expect reduced pSTAT3 to also lead to reduced Reg3b/g gene expression (classical pSTAT3 target genes), which is not the case. Finally, classically, it is thought that IL-23-IL-22 axis is responsible for the initial innate response to Citro (this induces pStat3 in epithelium) – the authors do not place their findings into this context and only relate it to IL-17A. This is important as the authors see clear effects such as weightloss at the initial stage.

**Response: Thank you for your comments and providing the valuable feedback on our manuscript. We appreciate the insightful suggestions for improvement. Regarding the concern about the lack of data demonstrating the success of the bone marrow transplant and its success rate,** **we designed distinct probes targeting both wild-type and knockout alleles of JMJD2D, and assessed the copy number of the murine peripheral blood genomic DNA using digital PCR. The efficiency of bone marrow transplantation was calculated to exceed 90%. We provided detailed data in Supplementary Figure S1, which emphasized the significance of these additional findings in supporting our research conclusions.**

**We also appreciate your suggestion to evaluate the proposed mechanisms, particularly the effect on pSTAT3 in the *in vivo* experiments. We agree that further *in vivo* studies would provide a more comprehensive understanding of the mechanisms involved, we therefore detected the phosphorylation of STAT3 in the colonic epithelial cells of WT and JMJD2D^-/-^ mice on days 7 and 14 after *C. rodentium* infection, which were presented in Supplementary Figure S3B. The results showed that STAT3 phosphorylation was impaired in the colonic epithelial cells of JMJD2D^-/-^ mice after *C. rodentium* infection.**

**Regarding your observation about the unexpected gene expression pattern of** **Reg3b/g despite reduced pSTAT3 levels, we acknowledge the discrepancy and understand the importance of addressing this inconsistency. In our revised manuscript,** **we treated wild-type and JMJD2D-knockdown CMT93 cells with IL-22 and then examined the expression of JMJD2D, p-STAT3, and Reg3b/g. As shown in Supplementary Figure S4C&D, JMJD2D knockdown reduced IL-22-induced STAT3 phosphorylation and Reg3beta expression (the expression of Reg3g in CMT93 cells is very low, we can hardly detect its expression by qRT-PCR). We also detected** **the recruitment of STAT3 on Reg3beta promoter in wild-type and JMJD2D-knockdown CMT93 cells without or with IL-22 treatment. As shown in Supplementary Figure S4E,** **JMJD2D knockdown reduced the recruitment of STAT3 on the Reg3beta promoter without or with IL-22 treatment. These data suggest that JMJD2D may promote Reg3beta expression by enhancing IL-22-induced STAT3 activation. However, in our current study, we did not observe significant changes in the expression of reg3b/g at 7 and 14 days after *C. rodentium* infection. This may be attributed to the peak expression of IL-22 occurring on the third day after infection (Ref. PMID: 18264109), while differences in reg3b/g expression manifest in the early stages following infection. Therefore, the question you raised, combined with our *in vitro* experimental studies in revised manuscript,** **suggest that JMJD2D may play a crucial regulatory role in the IL-22-STAT3-reg3b/g-mediated damage repair. However,** **the specific role and molecular mechanisms remain to be elucidated, and we propose this as a new topic for future investigation.** **Our current study mainly focuses on the role of JMJD2D in the regulation of STAT3-IL-17F-mediated β-defensin expression for bacterial clearance.**

**Part II – Major Issues: Key Experiments Required for Acceptance**
Please use this section to detail the key new experiments or modifications of existing experiments that should be absolutely required to validate study conclusions.

Generally, there should be no more than 3 such required experiments or major modifications for a "Major Revision" recommendation. If more than 3 experiments are necessary to validate the study conclusions, then you are encouraged to recommend "Reject".

Reviewer #1: n/a

Reviewer #2: (No Response)

Reviewer #3: To me there are 3 aspects that needs to be addressed:

- Confirmation of how successful the BM transplant was.

**Response: Thank you for raising the important question regarding the confirmation of the success of the bone marrow (BM) transplant in our study. We monitored the engraftment of donor-derived cells into the recipient's bone marrow by designing distinct probes targeting both wild-type and knockout alleles of JMJD2D, and assessed the copy number of the murine peripheral blood genomic DNA using digital PCR. The averaged efficiency of bone marrow transplantation was calculated to exceed 90%. We provided detailed data in** **Supplementary Figure S1, which emphasized the significance of these additional findings in supporting our research conclusions.**

- There needs to be a distinction between the ‘regenerative’ phenotype from the previous paper and the antimicrobial B-defensin phenotype proposed here in this manuscript (i.e. the impaired regeneration could also drive the inflammation/citro phenotype). What happens to the initial IL-23/IL-22 axis in these animals? IL-22 is thought to induce crypt hyperplasia (regenerative response), is this impaired (and this is causing the phenotype?)? To test, IL23/IL22 can be measured by qPCR, and Ki67 stain can define regenerative crypt hyperplasia response.

**Response: Thank you for your insightful comments and suggestions regarding the need to distinguish between the regenerative phenotype described in the previous paper and the antimicrobial β-defensin phenotype proposed in our manuscript. To address this concern,** **we detected the expression levels of IL-22 and IL-23 in the colons and isolated intestinal epithelial cells by qPCR. As shown in supplementary Figure S4A,** **no significant differences were observed in the expression of IL-22 and IL-23 in the colons of wild-type and JMJD2D^-/-^ mice on days 7 and 14 after *C. rodentium* infection. On day 14 after *C. rodentium* infection, JMJD2D deficiency led to impaired IL-22 expression in colonic epithelial cells. No significant difference was observed in IL-23 expression in colonic epithelial cells of wild-type and JMJD2D^-/-^ mice infected with *C. rodentium*. Additionally, we employed Ki67 staining to define the regenerative crypt hyperplasia response. The results revealed comparable numbers of Ki67+ cells per crypt between wild-type mice and JMJD2D^-/-^ mice without or with C. rodentium infection as shown in supplementary Figure S4B.** **This may be attributed to the peak expression of IL-22 occurring on the third day after infection (Ref. PMID: 18264109), while differences in regenerative response occurred in the early stages following infection. Thank you for bringing these important points to our attention. By incorporating these additional experiments, we aim to elucidate the relationship between impaired regeneration and the antimicrobial β-defensin phenotype. This will allow for a more comprehensive understanding of the mechanisms underlying the phenotype described in our manuscript and its potential association with the IL-23/IL-22 axis and regenerative responses.**

- The proposed mechanism is via STAT3 – however, this needs to be verified in vivo: What are pSTAT3 levels in vivo after infection. As other pSTAT3 targets (Reg3b/g) are not altered in the KO, why/how is IL17F specifically affected/tuned. pSTAT3 staining or by western should be performed (of infected animals to determine the response) to determine that this is the mechanism by which IL17F is controlled in vivo after infection.

**Response: Thank you for your insightful comments and suggestions regarding the proposed mechanism involving STAT3 in our manuscript. We acknowledge the importance of verifying the involvement of STAT3 *in vivo*, particularly regarding its phosphorylation status (pSTAT3 levels) after infection. To address this, we conducted western blot assays to assess pSTAT3 levels *in vivo* following infection. The results showed that STAT3 phosphorylation was impaired in the colonic epithelial cells of JMJD2D^-/-^ mice on days 7 and 14 after *C. rodentium* infection as shown in supplementary Figure S3B.**

**Furthermore, we appreciate your point regarding the discrepancy in the regulation of IL17F compared to other pSTAT3 targets such as Reg3b/g in the knockout (KO) animals. To address this, we treated wild-type and JMJD2D-knockdown CMT93 cells with IL-22 and then examined the expression of JMJD2D, p-STAT3, and Reg3b/g. As shown in Supplementary Figure S4C&D, JMJD2D knockdown reduced IL-22-induced STAT3 phosphorylation and Reg3beta expression (the expression of Reg3g in CMT93 cells is very low, we can hardly detect its expression by qRT-PCR). We also detected the recruitment of STAT3 on Reg3beta promoter in wild-type and JMJD2D-knockdown CMT93 cells without or with IL-22 treatment. As shown in Supplementary Figure S4E, JMJD2D knockdown reduced the recruitment of STAT3 on the Reg3beta promoter without or with IL-22 treatment. These data suggest that JMJD2D may play a crucial regulatory role in the IL-22-STAT3-mediated Reg3beta expression. However, the specific role and molecular mechanisms remain to be elucidated, and we propose this as a new topic for future investigation.**

**Part III – Minor Issues: Editorial and Data Presentation Modifications**
Please use this section for editorial suggestions as well as relatively minor modifications of existing data that would enhance clarity.

Reviewer #1: 1. Figure 3G, it is IL-17F protein level, not mRNA level.

**Response: Thank you for pointing out the mistake in Figure 3G. We appreciate your attention to detail. We have corrected that Figure 3G depicts the protein levels of IL-17F, not mRNA levels. We apologize for any confusion this may have caused.**

2. Also confirm the IL-17Ra and IL-17RC are mRNA levels or protein levels.

**Response: Thank you for your inquiry regarding the nature of the data presented for IL-17Ra and IL-17RC in our manuscript. We would like to confirm that the data presented for IL-17Ra and IL-17RC in Figure3 to mRNA levels, not protein levels. We apologize for any confusion that may have arisen regarding this matter. Your attention to detail is appreciated, and we thank you for bringing this to our attention.**

3. Reference #35 miss the journal name. Authors need to go over carefully all the references to make sure the information is intact.

**Response: Thank you for bringing this to our attention regarding Reference #35. We apologize for the oversight in missing the journal name for this reference. We will thoroughly review all references cited in our manuscript to ensure that the information is complete and accurate. Thank you for your diligence in reviewing our manuscript, and we appreciate the opportunity to correct this error.**

4. It is unclear how JMJD2D increases STAT3 phosphorylation.

**Response: Thank you for your comment regarding the unclear mechanism by which JMJD2D increases STAT3 phosphorylation. We acknowledge the need for clarity in explaining the mechanism underlying the observed increase in STAT3 phosphorylation mediated by JMJD2D.** **One possible mechanism could involve JMJD2D-mediated regulation of upstream signaling pathways that converge on STAT3 phosphorylation.** **Our recent study (PMID: 35027670) has demonstrated that JMJD2D can elevate the IFNGR1 expression, which modulated STAT3 phosphorylation in CMT93 cells. Thank you for highlighting this issue, and we will ensure that it is adequately addressed in our revised manuscript.**

Reviewer #2: Given that the authors performed only targeted gene expression and chromatin analysis, they should tone down the discussion a bit when they write (line 487 onwards): “ Thus, higher C. rodentium burdens in JMJD2D-/- mice are caused by reduced expression of β-defensin-1, β-defensin-3, and β-defensin-4 after C. rodentium infection, which lead to more severe colonic injury ” by rephrasing, e.g., “Thus, we propose that higher C. rodentium burdens in JMJD2D-/- mice are caused by a reduced innate immunity response, such as expression of β-defensin-1, β-defensin-3, and β-defensin-4 after C. rodentium infection, which lead to more severe colonic injury” or similar. There may be additional protective pathways that depend on JMJD2D that the authors simply have not screened for.

**Response: Thank you for your thoughtful feedback on our manuscript regarding the discussion on the potential mechanisms underlying the observed differences in *C. rodentium* burdens between JMJD2D^-/-^ mice and wild-type mice. We appreciate your suggestion to tone down the discussion and acknowledge the limitations of our study, particularly regarding the targeted gene expression and chromatin analysis performed. We agree that our findings may not capture all potential protective pathways dependent on JMJD2D, and it would be more appropriate to phrase our conclusions in a more cautious manner. We have revised the discussion section accordingly, acknowledging that our study focused on a subset of potential protective pathways and that additional pathways may exist that were not investigated in our study.**

Minor comments:
Line 33, abstract: “The enteric pathogen Citrobacter rodentium (C. rodentium) model was used to mimics clinical colonic infection.” Should be “to mimic”…
**Response: Thank you for pointing out the grammatical error in line 33 of the abstract. We appreciate your attention to detail and we have revised the sentence.**

Line 101: “IL-17F.(11)”, the reference should come before the period/full stop (.).

**Response: Thank you for catching that error in the reference formatting in line 101. Your attention to detail is much appreciated. We have corrected the formatting to ensure that the reference appears before the period/full stop, as follows: "IL-17F (11)."**

Reviewer #3: (No Response)
